# Prognostic Significance of the Tumor-Stromal Ratio in Invasive Breast Cancer and a Proposal of a New Ts-TNM Staging System

**DOI:** 10.1155/2020/9050631

**Published:** 2020-04-21

**Authors:** Qian Xu, Jing-Ping Yuan, Yuan-Yuan Chen, Hong-Yan Zhang, Lin-Wei Wang, Bin Xiong

**Affiliations:** ^1^Hubei Key Laboratory of Tumor Biological Behaviors, Hubei Cancer Clinical Study Center, Zhongnan Hospital of Wuhan University, 4300711 Wuhan, China; ^2^Department of Gastrointestinal Surgery, Zhongnan Hospital of Wuhan University, Wuhan, China; ^3^Department of Pathology, Renmin Hospital of Wuhan University, 430060 Wuhan, China; ^4^Department of Radiation and Medical Oncology, Zhongnan Hospital of Wuhan University, Wuhan, China

## Abstract

**Background:**

Previous studies have demonstrated that the tumor-stromal ratio (TSR) was an independent prognostic factor in several types of carcinomas. This study aimed at exploring the prognostic significance of the TSR in invasive breast cancer using immunohistochemistry (IHC)-stained tissue microarrays (TMAs) and integrating the TSR into the traditional tumor-node-metastasis (TNM) staging system.

**Methods:**

The prepared 7 TMAs containing 240 patients with 480 invasive BC specimens were stained with cytokeratin (CK) by the IHC staining method. The ratio of tumor cells and stromal cells was visually assessed. TSR > 1 and TSR ≤ 1 were categorized as the high TSR (low stroma) and low TSR (high stroma) groups, respectively, and the prognostic value of the TSR at 5-year disease-free survival (5-DFS) was analyzed. A new Ts-TNM (tumor stroma-tumor-node-metastasis) staging system was established and assessed.

**Results:**

IHC staining of CK could specifically label tumor cells with clear contrast, making it easy to manually assess TSR. High TSR (low stroma) and low TSR (high stroma) were observed in 52.5% (*n* = 126) and 47.5 (*n* = 114) of the cases, according to the division of value 1. A Kaplan–Meier analysis showed that patients in the low TSR group had a worse 5-DFS compared with patients in the high TSR group (*P*=0.022). Multivariable analysis indicated that the T stage (*P*=0.014), N status (*P* < 0.001), histological grade (*P* < 0.001), estrogen receptor status (*P*=0.015), and TSR (*P*=0.011) were independent prognostic factors of invasive BC patients. The new Ts-TNM staging system combining TSR, tumor staging, lymph node status, and metastasis staging was established. The receiver operating characteristic (ROC) curve analysis demonstrated that the ability of the Ts-TNM staging system to predict recurrence was not lower than that of the TNM staging system.

**Conclusions:**

This study confirms that the TSR is a prognostic indicator for invasive breast cancer. The Ts-TNM staging system containing stromal and tumor information may optimize risk stratification for invasive breast cancer.

## 1. Introduction

Breast cancer (BC) is the leading cause of cancer deaths among females in both developed and developing countries. With an estimated 1.6 million cases and 520,000 deaths every year, breast cancer alone accounts for 14% of all cancer deaths among females [1, 2]. Although considerable improvements have been achieved over the past few decades because of advancements in screening tools and comprehensive therapies for BC, cancer recurrence and distant metastasis still occurs in a proportion of patients [3, 4]. More prognostic factors are needed to optimize invasive breast cancer risk stratification to guide precise treatment.

Currently, the tumor-node-metastasis (TNM) staging system is the most frequently used classification criteria to determine the clinical stages of cancer, predict prognosis, and guide treatment strategies [5, 6]. With improvements in health consciousness and detection tools, patients in the early TNM stage have become the main part of BC. However, for patients with both N and M statuses negative, this staging system can only predict the risk of recurrence and metastasis to a certain degree [7]. Furthermore, the TNM system mainly focuses on the tumor's biological behaviors (tumor growth, lymph node invasion, and metastasis), and insufficient attention is paid to the nontumor factors that affect tumor progression [8, 9]. Therefore, it is imperative to develop a more accurate classification system by integrating the new and easily available prognostic factors into the traditional TNM staging system.

Recently, accumulating evidence suggests that tumor progression and metastasis are not only affected by biological behaviors of cancer cells but also by tumor microenvironments (TMEs), which are defined as the bidirectional interactions between tumor cells, stromal cells, and cellular elements [10, 11]. Tumor stroma, as an important component of TME, promotes tumor progression through production of various nutrition, growth factors, chemokines, and cytokines [12–14]. As a result, the tumor-stromal ratio (TSR), a new parameter that represents the proportion of tumor-associated stroma, was introduced to the field of cancer research [15]. Previous studies have demonstrated that the TSR is a new prognostic factor in cases of colon carcinoma [16], rectal adenocarcinoma [17], hepatocellular carcinoma [18], non-small cell lung cancer [19], gallbladder cancer [20], and breast cancer [21]. Currently, the TSR is largely assessed in the hematoxylin-eosin (HE) staining section, which may not accurately identify the boundary of tumor nests (TNs) due to low contrast between tumor and stroma and is unsuitable for large sample detection, computer recognition, and automatic analysis. As a result, we use tissue microarrays (TMAs) containing BC specimens, which were stained with cytokeratin (CK) in our previous study, to specifically label tumor cells [7].

This study aimed at exploring the prognostic value of the TSR in invasive BC using CK-stained TMAs. Furthermore, a new staging system combining TSR, tumor staging, lymph node staining, and metastasis is established to optimize risk stratification for invasive BC.

## 2. Materials and Methods

### 2.1. Patients and Specimens

Our center has established a clinical database of BC, which has been the data source of several clinical and translational studies [22, 23]. From the database, 240 invasive BC specimens were selected, and TMAs were prepared based on a clearly set criterion. Major clinicopathologic characteristics including age, menopausal status, histological type, *T* stage, *N* status, estrogen receptor (ER) status, and HER2 gene status of these patients were summarized. TNM staging and histological grading were determined according to the 8th edition of the UICC/AJCC TNM classification [24] and WHO histological grading [25]. The failure event of BC patients was locoregional recurrence, metastatic recurrence, or death. The 5-year disease-free survival (5-DFS) was used as the primary endpoint. Approval of the study protocol was obtained from the Institutional Ethics Committee of Zhongnan Hospital of Wuhan University. The study was undertaken according to the ethical standards of the World Medical Association Declaration of Helsinki.

### 2.2. Tissue Microarrays Construction

TMAs were prepared using standard procedures in collaboration with Shanghai Outdo Biotech Co. Ltd. (Shanghai, China), as previously described [26]. All specimens were stained with hematoxylin and eosin, and the most invasive tumor areas containing both tumor cells and tumor stroma were identified. Corresponding areas were marked in the paraffin blocks. From two marked areas of each paraffin block, two cores were taken using punch cores and deposited into the tissue microarray block with 70 cylinders. Then, seven TMAs with 480 cores were constructed. Duplicates of cylinders were included in each specimen to ensure reproducibility and homogenous staining of the slides.

### 2.3. IHC Staining of CK

IHC staining of CK was performed in our previous study [7]. First, TMAs were heated at 60°C for 2 h, immersed in dimethylbenzene for 15 min to deparaffinizing, and rehydrated in a series of alcohol. Then, the slides were pretreated in 0.01 mol/L citrate buffer (pH 6.0) and heated in a microwave oven (95°C) for 15 min. After cooling at room temperature, TMAs were blocked with 0.03% hydrogen peroxide methanol for 10 min and 2% bovine serum albumin (BSA) for 20 min to decrease background intensity. Every slide was treated overnight at 4°C with 250 *μ*l mouse anti-human CK monoclonal antibody (AE1/AE3, dilution 1 : 100, ZSGB-BIO, Beijing, China) and incubated with corresponding secondary antibody (dilution 1 : 250) for 30 min at 37°C. DAB (dilution 1 : 500, DAKO, Denmark) was then added and reacted for 2 min, and the samples were counterstained with hematoxylin and sealed with resin mount. Furthermore, HE-stained invasive BC sections were selected to make a comparison.

### 2.4. Assessment of TSR

The TSR was defined as the ratio of the tumor area to stromal area under a microscope. Principles of the TSR scoring in this study were applied according to the previously described criteria [27]. Compartments, including necrosis, microvessels, inflammation, and mucus-forming tumor tissue, were excluded. When the whole field of the microscope image was not filled with tumor tissue, areas that did not contain any tumor tissue would also be excluded. Two researchers assessed the tumor-stromal ratio independently using a 10 × objective lens. The field of the highest stromal percentage from the two cores of each case were considered crucial. Disagreement on the results was resolved by consensus. A third expert observer made the determination when no consensus could be reached. Concordance calculation and Cohen's kappa coefficient were used to assess agreement between the two independent observers in categorizing the TSR as high TSR or low TSR. Furthermore, the number of cases was noted, for which the third observer was consulted. According to previous studies [20, 28], a 50% cutoff point was usually selected to divide patients into stroma-low (proportion of stroma <50%) and stroma-high (proportion of stroma ≥50%) groups. As the TSR was defined as the ratio of the tumor area to stromal area under a microscope, the cutoff point of the TSR was selected as 1 (proportion of stroma = 50% equals to TSR = 1). TSR > 1 and TSR ≤ 1 were categorized as high TSR (low stroma) and low TSR (high stroma) groups, respectively.

### 2.5. Statistical Analysis

Statistical analysis was performed using IBM SPSS statistics (version 23.0 for Windows). The correlation between the TSR and other clinicopathologic factors was measured using the chi-squared test or Fisher's exact test. The Kaplan–Meier method and the log-rank test were performed to analyze five-year disease-free survival (5-DFS). Unadjusted HRs (hazard ratios) and 95% CIs (confidence intervals) of TSR for 5-DFS in each subgroup were calculated using a Cox proportional hazards model. Univariable and multivariable survival analyses were performed by the Cox proportional hazards method. The proportional hazard assumption was tested based on Schoenfeld residuals. Receiver operating characteristic (ROC) curve analysis was applied to determine the discriminatory ability of the tumor staging system. Two-sided *P* < 0.05 was considered statistically significant.

## 3. Results

### 3.1. IHC Staining Results in TMAs

**Figure 1:**
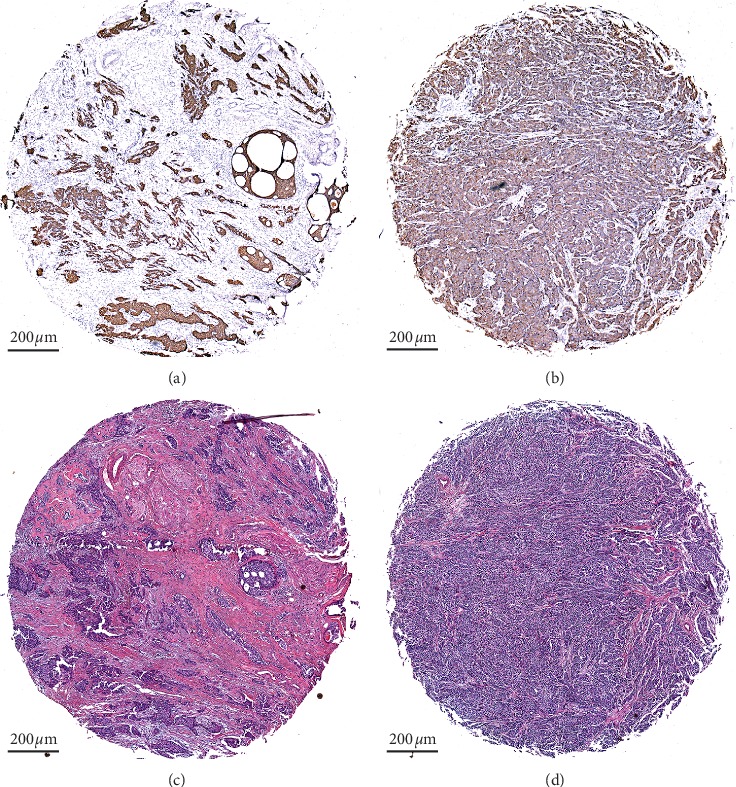
IHC staining and HE staining results in TMAs. IHC staining of CK could specifically label tumor areas with clear contrast (a, b). The differentiation of tumor and stroma in HE staining was not as clear (c, d). Examples of low TSR (high stroma) (a, c); examples of high TSR (low stroma) (b, d). HE,hematoxylin-eosin; IHC, immunohistochemistry; CK, cytokeratin; TSR, tumor-stromal ratio.

Typical examples of low TSR (high stroma) and high TSR (low stroma) cores in IHC staining and HE staining are shown in Figure 1. Figures 1(a) and 1(c) show the staining of specimens from the same patient with a low TSR, and Figures 1(b) and 1(d) show the staining of specimens from the same patient with a high TSR. After IHC staining, there was a strong color contrast of brown tumor cells and off-white tumor stroma (Figures 1(a) and 1(b)). By contrast, the differentiation of tumor and stroma in HE staining was not as clear as IHC staining. This may not clearly reveal the edge of all tumor nests (Figures 1(c) and 1(d)).

### 3.2. Evaluation of Tumor-Stromal Ratio

According to the definition of the TSR, BC patients were categorized into the high TSR (low stroma) and low TSR (high stroma) groups with 1 as the dividing value. Among 240 specimens, 52.5% were determined as the high TSR and 47.5% as the low TSR. In *n* = 28 (12%) cases, no consensus could be reached and the TSR was determined by the third observer. Concordance was 88.0% and Cohen's kappa value was 0.77, which reflected good agreement with TSR assessment.

### 3.3. Correlation between TSR and Major Clinical Characteristics

The prepared 7 tissue microarrays (TMAs) contained 240 invasive BC specimens. The age of the selected patients ranged from 29 to 78 years (median, 48 years) at the date of surgery.  Table 1 listed the major clinicopathological characteristics including age, menopausal status, histological type, *T* stage, *N* status, ER status, and HER2 gene status grouped by tumor-stroma ratio. The TSR correlated with the histological type (*P*=0.044) but not with age (*P*=0.636), menopausal status (*P*=0.927), *T* stage (*P*=0.966), *N* status (*P*=0.327), histological grade (*P*=0.302), ER status (*P*=0.164), and HER2 gene status (*P*=0.943) (Table 1).

### 3.4. Prognosis of BC Patients according to TSR

**Figure 2:**
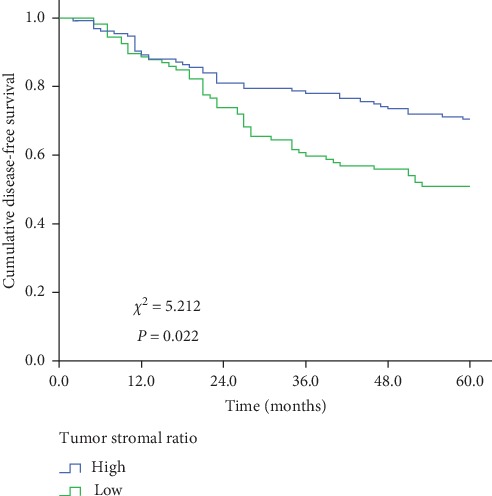
Differences between Kaplan–Meier plots for disease-free survival in each group calculated by the log-rank test. Low TSR (high stroma) was associated with worse 5-year disease-free survival (*χ*^2^ = 5.212, *P*=0.022). BC = breast cancer; TS = tumor-stromal ratio.

For 240 invasive BC patients, the 5-year disease-free survival rate was 62.0%. Traditional factors including *T* stage, *N* status, histological grade, histological type, ER status, HER2 gene status, and menopausal status were associated with invasive BC patients' 5-DFS (*P* < 0.05 for all) (Supplementary Table 1). The Kaplan–Meier survival curve for high- and low-TSR patients are shown in Figure 2. The 5-DFS in high-TSR (low stroma) and low-TSR (high stroma) groups were 69.0% and 54.3%, respectively, and the difference is statistically significant (*χ*^2^ = 5.212, *P*=0.022). The result suggested that the tumor-stromal ratio may be a prognostic parameter for invasive BC, and more stroma in tumor tissues indicated worse prognosis of BC patients.

### 3.5. Subgroup Analysis of the TSR for Association with 5-DFS

**Figure 3:**
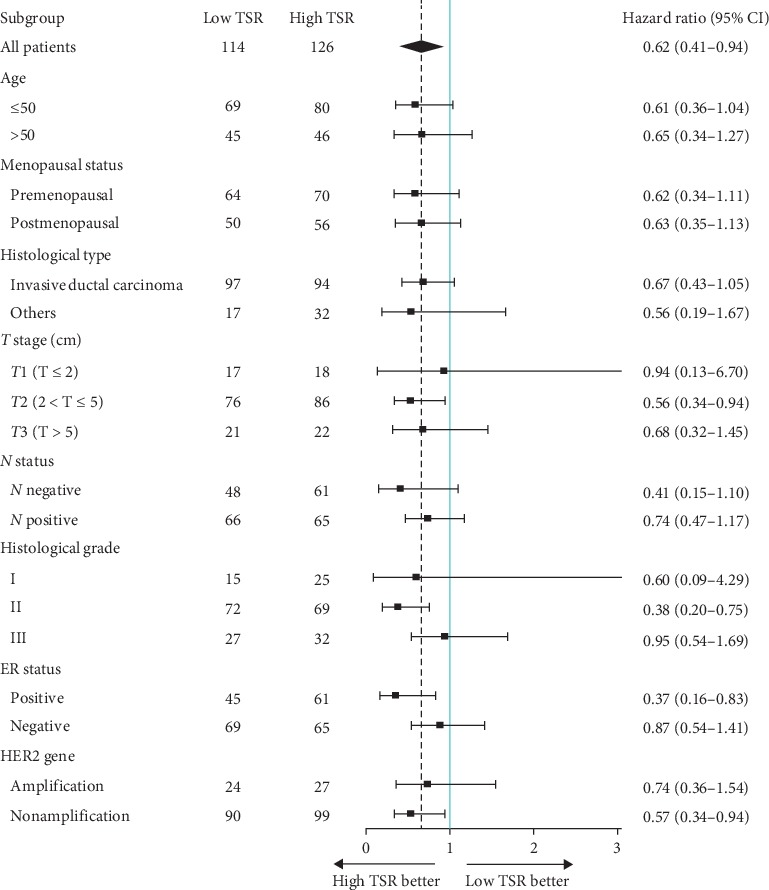
Forest plots of the TSR for association with 5-DFS in each subgroup. The dashed line showed the hazard ratio of 0.62 in all patients. CI = confidence interval.

The prognostic value of the TSR for 5-DFS was analyzed in each subgroup (Figure 3). High TSR (low stroma) was associated with improved 5-DFS in all patients (HR 0.621; 95% CI 0.410–0.941; *P*=0.025). Subgroup analysis revealed that the TSR was significantly associated with 5-DFS in T2 (HR 0.562; 95% CI 0.336–0.940; *P*=0.028), histological grade II (HR 0.383; 95% CI 0.195–0.754; *P*=0.006), ER status positive (HR 0.366; 95% CI 0.162–0.829; *P*=0.016) and HER2 gene nonamplification groups (HR 0.565; 95% CI 0.340–0.939; *P*=0.028). Furthermore, an association between high TSR and improved 5-DFS was observed in subgroups such as age, menopausal status, *N* status, and histological type but was not statistically significant (*P* > 0.05). HR of the *T*1 stage and histological grade I groups had a very broad confidence interval, probably caused by the relatively small sample size or wide sample variability.

### 3.6. Univariable and Multivariable Analysis of the TSR and Other Parameters

To proceed to a deeper analysis, the Cox univariable and multivariable models were applied to analyze the correlation between clinicopathological parameters and 5-DFS. Univariable analysis demonstrated that the *T* stage (*P* < 0.001), *N* status (*P* < 0.001), histological grade (*P* < 0.001), ER status (*P* < 0.001), HER2 gene status (*P* < 0.001), and TSR (*P*=0.025) were of prognostic significance. Multivariable analysis identified the *T* stage (*P*=0.014), *N* status (*P* < 0.001), histological grade (*P* < 0.001), ER receptor status (*P*=0.015), and TSR (*P*=0.011) as independent prognostic factors (Table 2). HR of the TSR was 1.742 (95% CI, 1.137–2.669), which was lower than the *N* status and histological grade but higher than *T* stage, ER status and HER2 gene status.

### 3.7. Establishment and Prognostic Analysis of the Ts-TNM Staging System

To further investigate prognostic significance of the TSR, the TSR was integrated into the TNM staging system to establish a new Ts-TNM (tumor stroma tumor-node-metastasis) staging system. As the low-TSR (high stroma) group was associated with worsened disease-free survival, patients in the low-TSR group were defined as Ts1 and patients in the high-TSR group as Ts0. Patients of the low-TSR (Ts1) group in the TNM staging system were assigned to the next higher stage in the Ts-TNM staging system, and patients of the high-TSR (Ts0) in the TNM staging system remained at the same stage in the Ts-TNM staging system (Table 3). Based on this criterion, 9 patients of the low-TSR group in TNM stage I were assigned to Ts-TNM stage II, 65 patients of the low-TSR group in TNM stage II were assigned to Ts-TNM stage III, and 38 patients of the low-TSR group in stage III were assigned to Ts-TNM stage IV.

**Figure 4:**
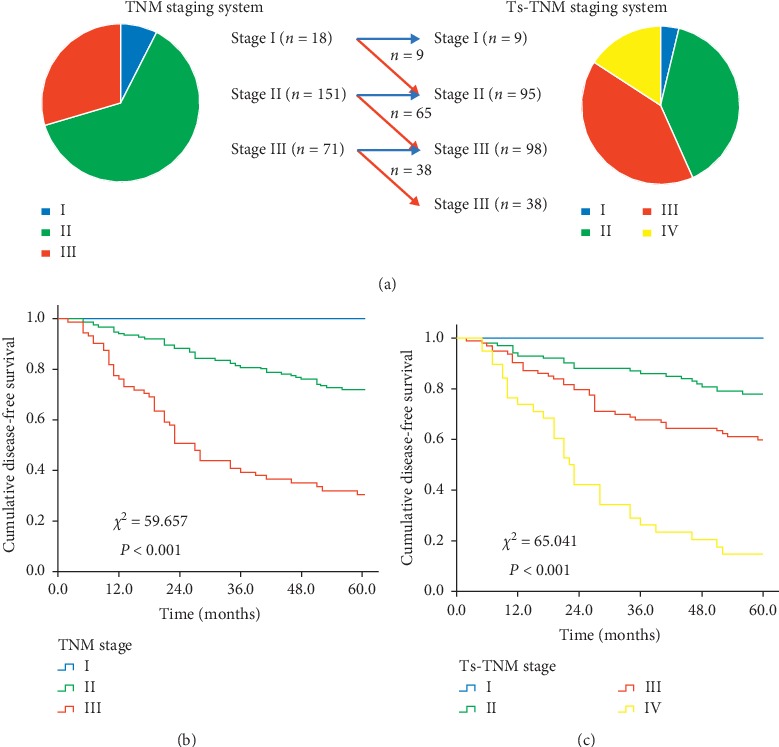
Patient distribution and prognostic analysis of the TNM and Ts-TNM staging system. Patients of low TSR (Ts1) in the TNM staging system were assigned to the next higher stage in the Ts-TNM staging system (red arrow), and patients of high TSR (Ts0) in the TNM staging system remained at the same stage in the Ts-TNM staging system (blue arrow) (Table 3). (a) Both TNM and Ts-TNM staging system can well distinguish BC patients into subgroups with different prognosis (b and c). TNM = tumor node metastasis; Ts-TNM = tumor-stroma tumor node metastasis; *T* = tumor; *N* = node.

A Kaplan–Meier survival analysis was applied to verify the ability of the TNM and Ts-TNM staging systems to stratify the risk of recurrence. The survival curve indicated that the TNM staging system can well distinguish BC patients into three subgroups with different prognoses (*χ*^2^ = 59.657, *P* < 0.001). Similarly, the Ts-TNM staging system can also appropriately distinguish BC patients into four subgroups with different prognoses (*χ*^2^ = 65.041, *P* < 0.001). The *χ*^2^ value of the TS-TNM staging system (*χ*^2^ = 65.041) was higher than that of the TNM staging system (*χ*^2^ = 59.657) (Figures 4(b) and 4(c)).

### 3.8. Comparison of the Predictive Value of the TNM and Ts-TNM Staging System

A ROC curve analysis was applied to compare the ability of the TNM and Ts-TNM staging systems to predict recurrence. The AIC value and Harrell's C value of the two staging systems were also calculated. The area under the curve (AUC) of the Ts-TNM staging system (AUC: 0.727; 95% CI: 0.661–0.794) was slightly larger than the AUC of the TNM staging system (AUC: 0.723; 95%CI: 0.657–0.790). The AIC value and Harrell's C value of the TNM staging system were 904.308 and 0.691, respectively, while those of the Ts-TNM staging system were 908.425 and 0.687, respectively. This demonstrates that the ability of the Ts-TNM staging system to predict recurrence was not lower than the TNM staging system's ability.

## 4. Discussion

Accumulating evidence has emphasized the significance of TME in tumor progression, and the importance of the TSR, as a new parameter which represents the amount of tumor-associated stroma, has been reported in different cancer types. Recent studies focused largely on the prognostic value of the TSR, as the high percentage of tumor stroma tends to be correlated with unfavorable prognosis [16, 17].

Reliable assessment is the basis to explore prognosis of the TSR. Currently, there are two methods to assess the TSR in the HE-stained section. One is visual eyeballing, a manual method with two-steps to determine the TSR [31]. First, observers select the most invasive tumor areas at low magnification. Then, fields containing tumor cells and tumor stroma are assessed at high magnification, and the estimate is recorded as the tumor-stromal ratio and scored per tenfold percentage. The other is a semiautomated point counting method developed by West et al. [32], and validated for breast cancer by Downey et al. [33]. Four-micrometer-thick HE-stained sections are scanned, and the most invasive tumor areas were selected. Subsequently, a total of 300 points are randomly inserted into the selected area. The histopathology is categorized as “tumor,” “stroma,” or “unclassified (necrosis, blood vessels, and inflammation).” The ultimate TSR is the number of points that are categorized as “tumor” divided by the number of points that are categorized as “stroma.” Scores are given per tenfold percentage.

These two methods have been applied in various studies [33–35]. Both methods assess the TSR using HE-stained histologic sections and could be easily implemented in routine pathology diagnostics. However, sometimes, the boundary of the tumor nests cannot be accurately identified because of the low contrast between the tumor and stroma, which may compromise the repeatability of the results and making it difficult to perform accurate recognition and analysis. As a result, IHC staining of the CK was applied to specifically label the tumor cells in our study. It resulted in a strong color contrast of marking tumor cells in brown and the tumor stroma in off-white. Moreover, it is well known that breast cancer is a heterogeneous disease. Distribution of carcinoma cells varied in different sampling sites. As a result, the most invasive tumor areas containing both tumor cells and the tumor stroma were identified and marked to construct TMAs. Two tumor cores were taken from each specimen, and the field with the highest stromal percentage from two cores was considered crucial. These two advantages make it more objective and efficient for observers to assess the TSR compared with other similar studies. More importantly, the utilization of the CK-stained TMAs may contribute to future potential large sample detections, computer recognitions, and automatic analyses.

So far, the TSR has been reported to be of prognostic value for BC in several studies. Kruijf et al. [21] demonstrated that early BC patients with stroma-rich tumors had a higher risk of relapse than those with stroma-poor tumors, especially in triple-negative breast cancer patients. Moorman et al. [36] also identified the TSR as a strong independent prognostic variable in triple-negative breast cancer patients. A study from the perioperative chemotherapy trial (POP trial, 10854) validates the prognostic value of the TSR in lymph node-negative premenopausal BC patients [34]. The prognostic value of the TSR in primary operable invasive ductal BC [35], estrogen receptor-positive BC [33], and inflammatory BC [37] was also confirmed. This study aimed at exploring the prognostic value of the TSR in invasive BC using CK-stained TMAs, and 240 invasive BC specimens were selected from an established clinical database. The characteristics of the patients in this study displayed similar features from central Chinese but is different from the general population of invasive breast cancer patients, in that the study population is younger in onset age and has less patients with lymph node negative, small tumor size, and ER positive [38, 39]. The lower rate of lymph node negative indicated more patients with aggressive BC, and the lower rate of ER positive indicated more patients disqualified for endocrine therapy. As a result, prognosis of the subjects is poorer than that of common patients with invasive breast cancer. In line with previous studies, this study revealed that invasive BC patients in the low-TSR group had a worse 5-DFS compared with patients in the high-TSR group, and the TSR was not associated with age, menopausal status, *T* stage, *N* status, histological grade, ER status, and HER2 gene status. Subgroup analysis revealed that the TSR was significantly associated with 5-DFS in T2, histological grade II, ER status positive, and HER2 gene status nonamplification groups. Multivariable analysis identified the *T* stage, *N* status, histological grade, hormone receptor status, and TSR as independent prognostic factors of invasive BC patients.

When the *N* and *M* statuses of patients is both negative, the TSR can provide information to predict the risk of recurrence and metastasis. As a result, we integrated the TSR into the traditional TNM staging system and established a new Ts-TNM staging system creatively in BC. It stratified 240 invasive BC patients into four subgroups (stages I, II, III, and IV) with different prognosis. The ROC analysis demonstrated that the ability of the Ts-TNM staging system to predict recurrence was not lower than the TNM staging system. Furthermore, the Ts-TNM staging system, combining tumor's biological behaviors (tumor size, lymph node spread, and distant metastasis) with stromal status (low TSR and high TSR), may be a new paradigm to encompass tumor heterogeneity. Furthermore, the TSR can be easily assessed in routine pathology diagnostics, which makes it feasible to perform Ts-TNM staging.

However, there are still some disadvantages and limitations in our study. First, this research is retrospective, and the sample capacity is relatively small, especially for the TNM stage I group. All 9 patients in the Ts-TNM stage I group did not have a recurrence after 5 years, which indicates that the prognostic value of the TSR for BC patients with TNM stage I remains uncertain. It will be valuable to conduct a prospective study with a larger sample for the TNM stage I group. Second, although TMAs are strictly constructed according to the criterion that only the most invasive tumor areas containing both tumor cells and tumor stroma are selected, not every core of TMAs can completely represent the optimal site to determine the TSR. More tumor cores taken from each specimen may reduce selection bias. Last, manual assessment of the TSR limits reliability of the results. A computer recognition and analysis software will greatly enhance efficiency, which may be a more optimal method for analyzing histological images.

## 5. Conclusion

In general, our study uses CK-stained TMAs and demonstrates that invasive BC patients of low TSR have poor prognoses. Furthermore, the Ts-TNM staging system combining the TSR, tumor staging, lymph node status, and metastasis staging can provide supplementary information in predicting the risk of recurrence and metastasis and may serve as a new paradigm to encompass tumor heterogeneity.

## Figures and Tables

**Table 1:** Relationship between TSR and major clinicopathological characteristics.

Characteristics	Total, *n* (%)	Low TSR, *n* (%)	High TSR, *n* (%)	*P* value
Age (years)				0.636
≤50	149 (62.1)	69 (60.5)	80 (63.5)	
>50	91 (37.9)	45 (39.5)	46 (36.5)	
Menopausal status				0.927
Premenopausal	134 (55.8)	64 (56.1)	70 (55.6)	
Postmenopausal	106 (44.2)	50 (43.9)	56 (44.4)	
Histological type				0.044
Invasive ductal carcinoma	191 (79.6)	97 (85.1)	94 (74.6)	
Others	49 (20.4)	17 (14.9)	32 (25.4)	
*T* stage (cm)				0.966
*T*1 (*T* ≤ 2)	35 (15.0)	17 (14.9)	18 (14.3)	
*T*2 (2 < *T* ≤ 5)	162 (67.5)	76 (66.7)	86 (68.2)	
*T*3 (*T* > 5)	43 (17.5)	21 (18.4)	22 (17.5)	
*N* status				0.327
*N* negative	109 (45.4)	48 (42.1)	61 (48.4)	
*N* positive	131 (54.6)	66 (57.9)	65 (51.6)	
Histological grade				0.302
I	40 (16.7)	15 (13.2)	25 (19.8)	
II	141 (58.8)	72 (63.2)	69 (54.8)	
III	59 (24.6)	27 (23.6)	32 (25.4)	
ER status^a^				0.164
Positive	106 (44.2)	45 (39.5)	61 (48.4)	
Negative	134 (55.8)	69 (60.5)	65 (51.6)	
HER2 gene^b^				0.943
Amplification	51 (21.3)	24 (21.0)	27 (21.4)	
Nonamplification	189 (78.7)	90 (79.0)	99 (78.6)	

^a^ER was determined by immunohistochemistry staining according to the guideline [29]; ^b^HER2 gene was determined by fluorescent in situ hybridization (FISH) according to the guideline [30]. BC, breast cancer; *T*, tumor; *N*, node; TSR, tumor-stromal ratio; ER, estrogen receptor; HER2, human epidermal growth factor receptor-2.

**Table 2:** Univariable and multivariable analysis of parameters associated with 5-DFS.

Parameters	Univariable analysis		Multivariable analysis	
	HR (95% CI)	*P* value	HR (95% CI)	*P* value
*T* stage	2.542 (1.753–3.686)	<0.001	1.583 (1.100–2.280)	0.014
*N* status	5.035 (2.966–8.545)	<0.001	3.948 (2.302–6.772)	<0.001
Histological grade	4.439 (3.063–6.433)	<0.001	2.825 (1.883–4.236)	<0.001
ER status	0.363 (0.237–0.555)	<0.001	0.567 (0.358–0.897)	0.015
HER2 gene	2.398 (1.541–3.733)	<0.001	1.614 (0.995–2.618)	0.053
TSR	1.610 (1.062–2.440)	0.025	1.742 (1.137–2.669)	0.011

*T*, tumor; *N*, node; TSR, tumor-stromal ratio; ER, estrogen receptor; HER2, human epidermal growth factor receptor-2.

**Table 3:** Definition of the TNM and Ts-TNM staging system.

TNM stage	*T*	*N*	*M*	TNM stage	Ts	Ts-TNM stage
I	*T*1	*N*0	*M*0	I	Ts0	I
II	*T*0	*N*1	*M*0		Ts1	II
	*T*1	*N*1	*M*0	II	Ts0	II
	*T*2	*N*0	*M*0		Ts1	III
	*T*2	*N*1	*M*0	III	Ts0	III
	*T*3	*N*0	*M*0		Ts1	IV
III	*T*0	*N*2	*M*0			
	*T*1	*N*2	*M*0			
	*T*2	*N*2	*M*0			
	*T*3	*N*1-2	*M*0			
	*T*4	*N*0–2	*M*0			
	Any *T*	*N*3	*M*0			

TNM, tumor node metastasis; Ts-TNM, tumor-stroma tumor node metastasis; *T*, tumor; *N*, node; Ts, tumor-stroma; Ts1, low TSR (high stroma); Ts0, high TSR (low stroma).

## Data Availability

Previously reported data on major clinicopathologic characteristics of patients were used to support this study and are available at 10.1016/j.biomaterials.2010.07.091. These prior studies (and datasets) are cited at relevant places within the text as references.

## Supplementary Materials

Supplementary Materials Supplementary Table 1: analysis of characteristics regarding 5-DFS.
