# Supplementary material for: Prognostic Significance of the Tumor-Stromal Ratio in Invasive Breast Cancer and a Proposal of a New Ts-TNM Staging System
Source: J Oncol. 2020 Apr 21;2020:9050631. doi: 10.1155/2020/9050631 (PMC7191412; doi:10.1155/2020/9050631)
Supplement: Supplementary Materials — Supplementary Table 1: analysis of characteristics regarding 5-DFS. [file 9050631.f1.pdf]

Supplementary Table 1 Analysis of characteristics regarding 5-DFS

Characteristics	Total (N=240)	No. of disease (%)	5-DFS rate (%)	P value
**Age (years)**				0.182
≤ 50	149 (62.1)	55 (60.4)	63.1	
> 50	91 (37.9)	36 (39.6)	60.4	
**Menopausal status**				<0.001
Premenopausal	134 (55.8)	45 (49.5)	66.4	
Postmenopausal	106 (44.2)	46 (50.5)	56.6	
**Histological type**				<0.001
Invasive ductal carcinoma	191 (79.6)	78 (85.7)	59.2	
Others	49 (20.4)	13 (14.3)	73.5	
**T stage (cm)**				<0.001
T1 (T ≤ 2)	35 (15.0)	4 (4.4)	88.6	
T2 (2 < T ≤ 5)	162 (67.5)	60 (65.9)	63.0	
T3 (T > 5)	43 (17.5)	27 (28.7)	37.2	
**N status**				<0.001
N negative	109 (45.4)	17 (18.7)	84.4	
N positive	131 (54.6)	74 (81.3)	43.5	
**Histological grade**				<0.001
I	40 (16.7)	4 (4.4)	90.0	
II	141 (58.8)	40 (43.9)	71.6	
III	59 (24.6)	47 (51.7)	20.3	
**ER status**				<0.001
Positive	106 (44.2)	25 (27.5)	76.4	
Negative	134 (55.8)	66 (72.5)	50.7	
**HER2 gene**				<0.001
Amplification	51 (21.3)	29 (31.9)	43.1	

Non-amplification	189 (78.7)	62 (68.1)	67.2

T: tumor; N: node; 5-DFS: 5-year disease free survival; ER: estrogen receptor; HER2: human epidermal growth factor receptor-2
